# Hepatoprotective Potential of *Caesalpinia crista* against Iron-Overload-Induced Liver Toxicity in Mice

**DOI:** 10.1155/2012/896341

**Published:** 2012-07-17

**Authors:** Rhitajit Sarkar, Bibhabasu Hazra, Nripendranath Mandal

**Affiliations:** Division of Molecular Medicine, Bose Institute, P-1/12 CIT Scheme VIIM, Kolkata 700054, India

## Abstract

The present study was carried out to evaluate the ameliorating effect of *Caesalpinia crista* Linn. (CCME) extract on iron-overload-induced liver injury. Iron overload was induced by intraperitoneal administration of iron dextran into mice. CCME attenuated the percentage increase in liver iron and serum ferritin levels when compared to control group. CCME also showed a dose-dependent inhibition of lipid peroxidation, protein oxidation, and liver fibrosis. The serum enzyme markers were found to be less, whereas enhanced levels of liver antioxidant enzymes were detected in CCME-treated group. In presence of CCME, the reductive release of ferritin iron was increased significantly. Furthermore, CCME exhibited DPPH radical scavenging and protection against Fe^2+^-mediated oxidative DNA damage. The current study confirmed the hepatoprotective effect of CCME against the model hepatotoxicant iron overload and the activity is likely related to its potent antioxidant and iron-chelating property.

## 1. Introduction

Liver is one of the largest organs in the human body and the main site for intense metabolism and excretion. It is involved with almost all the biochemical pathways to growth, fight against disease, nutrient supply, energy provision, and reproduction [1]. Hepatic damage is associated with distortion of these metabolic functions [2] and, sometimes, resulting in serious health problems. Hepatotoxicity is the most common finding in patients with iron overloading as liver is mainly the active storage site of iron in our body [3]. Iron, the most important transition element of the body, is found in functional forms in haemoglobin, myoglobin, the cytochromes, enzymes with iron sulphur complexes, and other iron-dependent enzymes [4]. Although an optimum level of iron is always maintained by the cells to balance between essentiality and toxicity, in some situations it is disrupted, resulting in iron overload which is associated to the oxidative stress induced disorders including anemia, heart failure, liver cirrhosis, fibrosis, diabetes, arthritis, depression, impotency, infertility, and cancer [5]. In all iron-overload-induced diseases, iron removal by iron chelation therapy is an effective life-saving strategy. The currently available iron-chelating agents used clinically are deferoxamine, 1,2-dimethyl-3-hydroxypyrid-4-one (deferiprone, L1), and deferasirox. However, such compounds show several side effects and limitations [6, 7] that direct towards the finding of a more effective and safe drug [8, 9] which may rise the therapeutic benefits for patients.

Phytoconstituents including phenolics and flavonoids are most important representatives to offer alleviation of hepatic ailments. It has been found that most of them are effective antioxidants [10, 11] and iron chelation is very important part of their antioxidant activity [12]. Thus, search for crude drugs of the plant origin with antioxidant activity has become a central focus of study of hepatoprotection. *Caesalpinia crista *Linn. (syn. *C. bonducella*[L.]Roxb.) (family-Fabaceae) is a large scandent prickly shrub widely distributed throughout the tropical and subtropical regions of Southeast Asia. In India, it is commonly known as *Katikaranja* or *Natakaranja* and used in different system of traditional medication for the treatment of diseases and ailments of human beings. Traditionally, in Ayurveda, various plant parts such as leaves, stem, root, seed, and oil are used as febrifugal, periodic, tonic, and vesicant for the treatment of gynaecological disorders, skin diseases, constipation, piles, and ulcers [13]. Phytochemical investigations of this plant have revealed the presence of several cassane- and norcassane-type diterpenes [14–18]. This plant has profound medicinal use and reported to have adaptogenic [19], anthelmintic [20], anti-inflammatory [21], antipyretic and analgesic [22, 23], antimalarial [24], antiamyloidogenic [25], antibacterial [26], antifilarial [27], antitumor [28], anticonvulsant [29], nootropic [30], immunomodulatory [31], hepatoprotective [32], anxiolytic [33], antidiabetic, and hypoglycemic activity [34]. Previously, 70% methanol extract of *Caesalpinia crista* (CCME) leaf has shown *in vitro* antioxidant and iron-chelating property and was found to be a rich source of phenolic and flavonoid compound [35]. Therefore, the present study was undertaken to assess whether the damage caused to liver by iron overload can be normalised by administration of CCME in mice.

## 2. Material and Methods

### 2.1. Chemicals

Iron dextran and guanidine hydrochloride were purchased from Sigma-Aldrich, USA. Trichloroacetic acid (TCA), nitro blue tetrazolium (NBT), reduced nicotinamide adenine dinucleotide (NADH), phenazinemethosulfate (PMS), ferrozine, glutathione reduced, bathophenanthrolinesulfonate disodium salt, Thiobarbituric acid (TBA), and 5,5′-dithiobis-2-nitrobenzoic acid (DTNB) were obtained from Sisco Research Laboratories Pvt. Ltd, Mumbai, India. Hydrogen peroxide, ammonium iron (II) sulfatehexahydrate [(NH_4_)_2_Fe(SO_4_)_2_6H_2_O], 1-chloro-2,4-dinitrobenzene (CDNB), chloramine-T, hydroxylamine hydrochloride, Dimethyl-4-aminobenzaldehyde, and 2,4-dinitro phenylhydrazine (DNPH) were obtained from Merck, Mumbai, India. Ferritin was purchased from MP Biomedicals, USA. Streptomycin sulphate was obtained from HiMedia Laboratories Pvt. Ltd, Mumbai, India. The standard oral iron-chelating drug, desirox, was obtained from Cipla Ltd., Kolkata, India.

### 2.2. Plant Material

The leaves of *Caesalpinia crista* (CC) were collected from the Bankura district of West Bengal, India. It was identified and authenticated by the Central Research Institute (Ayurveda), Kolkata, India, and a voucher specimen (CRHS 121/08) was deposited there.

### 2.3. Preparation of Plant Extract

The leaves of CC were dried at room temperature for 7 days, finely powdered and used for extraction. The powder (100 g) was mixed with 500 ml methanol : water (7 : 3) using a magnetic stirrer for 15 hours, then the mixture was centrifuged at 2850 ×g and the supernatant was decanted. The extraction was repeated again with the precipitated pellet. The supernatants were collected, concentrated in a rotary evaporator and lyophilized. The dried extract, denoted as CCME was stored at −20°C until use.

### 2.4. Animals

Male Swiss albino mice (20 ± 2 g) were purchased from Chittaranjan National Cancer Institute (CNCI), Kolkata, India, and were maintained under a constant 12 h dark/light cycle at an environmental temperature of 22 ± 2°C. The animals were provided with normal laboratory pellet diet and water ad libitum. Institutional animal ethics committee (IAEC) approved all experiments performed and care of the animals was taken as per the guidelines of the committee for the purpose of control and supervision of experiments on animals (CPCSEA), Ministry of Environment and Forest, Government of India.

### 2.5. *In Vitro* Study

#### 2.5.1. DPPH Radical Scavenging

The free radical scavenging activity of CCME was evaluated by 1,1-diphenyl-2-picrylhydrazyl (DPPH) using a standard method [36]. Briefly, the reaction mixture contains 0.05 ml of 1 mM DPPH solution, 0.5 ml of 99% ethanol, and 0.45 ml of sample and standard ascorbic acid at different concentrations. The solution was rapidly mixed and the reduction of DPPH was measured by reading the decrease in absorbance at 517 nm. All tests performed six times. Ascorbic acid was used as a reference compound.

#### 2.5.2. Oxidative DNA Damage

pUC18 plasmid DNA was used for DNA protection study by CCME, according to a previously described method [37] with minor modifications. In Hepes buffer (pH 7.2, 100 mM), FeSO_4_ solution (750 *μ*M), CCME of varying doses (0–30 *μ*g/ml), DNA (0.5 mg/ml), and water were added to make an initial reaction mixture. Finally, H_2_O_2_ solution (7.5 mM) was added to start the reaction. Desferal was used to stop the reaction after 10 min. 25 *μ*l of each reaction mixture was loaded in 1% agarose gel. After migration, the gel was stained with ethidium bromide and visualized in a UV transilluminator. The DNA bands were quantified through densitometry and the following formulae were used to calculate the percentage of protection:

(1) $$\begin{array}{c} \%\;\text{SC}=\left[1.4\times \frac{\text{SC}}{\text{OC}+\left(1.4\;\times \;\text{SC}\right)}\right]\times 100, \end{array}$$

where, SC is the supercoiled; OC is the open circular; 1.4 is the correction factor

(2) % protection   =100×[control SC  −  chelator SC  control SC  −  no  chelator SC−1].

The ability of the plant extract to protect the DNA supercoil can be expressed by the concentration of sample required for 50% protection, designated as the [*P*]_50_ value.

### 2.6. *In Vivo* Study

#### 2.6.1. Experimental Design

Thirty-six mice were divided into six groups containing six mice in each group. One group served as blank (B) and received normal saline only. The other five groups were given five doses (one dose every two days) of 100 mg/kg b.w. each, of iron dextran saline (i.p). One iron dextran group (C) received normal saline and other four groups were orally administered with 50 mg/kg b.w. (S50), 100 mg/kg b.w. (S100), 200 mg/kg b.w. (S200) plant extract, and 20 mg/kg b.w. desirox (D), respectively, for three consecutive 7 day periods, started from the day after the first iron dextran injection.

#### 2.6.2. Sample Collection and Tissue Preparation

Mice were fasted overnight after the experiment ended on the 21st day. They were anesthetized with ethyl ether and blood was collected by cardiac puncture. After the clotting of blood samples, sera were separated using cooling centrifuge and store at −80°C until analysis. The liver was dissected out and rinsed with ice-cold saline to eliminate the blood cells; half of them were cut, weighed, and homogenized in 10 volume of 0.1 M phosphate buffer (pH 7.4) containing 5 mM EDTA and 0.15 M NaCl, and centrifuged at 8000 g for 30 min at 4°C. The supernatant was collected and used for the assay of enzyme activities, protein oxidation, levels of hydroxyproline content, and lipid peroxidation products. A standard graph of BSA was prepared to estimate the protein concentration in the homogenate by Lowry method [38]. The other half of the liver samples were weighed and digested with equivolume (1 : 1) mixture of sulphuric acid and nitric acid and their iron content were analysed.

#### 2.6.3. Serum Markers

Alanine amino transferase (ALAT), aspartate amino transferase (ASAT), and billirubin in serum samples were measured using the commercial kits of Merck, Mumbai, India. Serum alkaline phosphatase (ALP) was estimated using the kit supplied by Sentinel diagnostics, Italy.

#### 2.6.4. Antioxidant Enzymes

Superoxide dismutase (SOD) was assayed by measuring the inhibition of the formation of blue colored formazan at 560 nm [39]. Catalase (CAT) activity was measured by following the decomposition of H_2_O_2_ over time at 240 nm [40]. Glutathione-S-transferase (GST) was determined based on the formation of GSH-CDNB conjugate and increase in the absorbance at 340 nm [41]. A spectrophotometric method was used to measure reduced glutathione (GSH) level at 412 nm [42].

#### 2.6.5. Biochemical Parameters

The lipid peroxide levels in liver homogenates were measured in terms of thiobarbituric acid reactive substances (TBARS) as an index of malondialdehyde accumulation [43]. As a marker of protein oxidation, protein carbonyl contents were estimated spectrophotometrically by DNPH method [44]. Briefly, 50 *μ*l streptomycin sulphate (10% w/v) was added to 450 *μ*l homogenate samples and then centrifuged at 2800 g for 15 minutes. The supernatant (200 *μ*l) was incubated with the same volume of 10 mM DNPH in 2 M HCl at room temperature for 20 mins. After the reaction was completed, 10% cold TCA was added to precipitate the proteins and the precipitates were washed with ethyl acetate-ethanol mixture (1 : 1) for three times to remove unreacted DNPH. The final protein pellet was dissolved in 1 ml of 6 M guanidine hydrochloride solution and the absorbance was measured at 370 nm, using the molar extinction coefficient of DNPH, *ε* = 2.2∗10^−4^ M^−1^ cm^−1^. The measurement of hydroxyproline content in the liver allows the actual quantitation of collagen content which is an important marker of liver fibrosis. Liver samples were hydrolyzed in 6 M HCl and hydroxyproline was measured by Ehrlich's solution [45]. A standard curve (*R* ^²^ = 0.9907) of 4-hydroxy-L-proline was prepared and results were calculated after taking absorbances at 558 nm. Total hydroxyproline content in each sample was multiplied by a factor of 7.69 to determine the collagen content [46]. Results are expressed as milligrams of collagen per liver (wet weight).

#### 2.6.6. Histopathological Analysis

The liver samples were excised, washed with normal saline, and processed separately for histological study. Initially, the material was fixed in 10% buffered neutral formalin for 48 h. A paraffin-embedding technique was carried out and sections were taken at 5 *μ*m thickness, stained with hematoxylin and eosin, and examined microscopically for histopathological changes.

#### 2.6.7. Liver Iron and Serum Ferritin

Liver iron was measured according to a formerly reported colorimetric method [47]. Samples were incubated with bathophenanthrolinesulfonate for 30 min at 37°C and absorbances were read at 535 nm. Serum ferritin levels were measured using enzyme-linked immunosorbent assay kit (from Monobind Inc., USA) according to the manufacturer's instructions.

#### 2.6.8. Iron Release from Ferritin

As previously described, iron reduction and release was determined spectrophotometrically [48]. The ferrous chelator, ferrozine, was used as a chromophore for this assay. The reaction mixture (3 ml final volume) contained 200 *μ*g ferritin, 500 *μ*M ferrozine, in 50 mM pH 7.0 phosphate buffer. Reaction was induced by the addition of 500 *μ*l plant extracts of different concentrations and alteration in absorbance was measured continuously at 560 nm for 20 min. A cuvette containing ferritin, ferrozine, and phosphate buffer but lacking plant extract was used as the reference solution.

### 2.7. Statistical Analysis

All data are reported as the mean ± SD of six measurements. Statistical analysis was performed using KyPlot version 2.0 beta 15 (32 bit) and Origin professional 6.0. Comparisons among groups were made according to pair *t*-test. In all analyses, a *P*-value of <0.05 was considered significant.

## 3. Results and Discussion

### 3.1. *In Vitro* Study

#### 3.1.1. DPPH Scavenging

**Figure 1:**
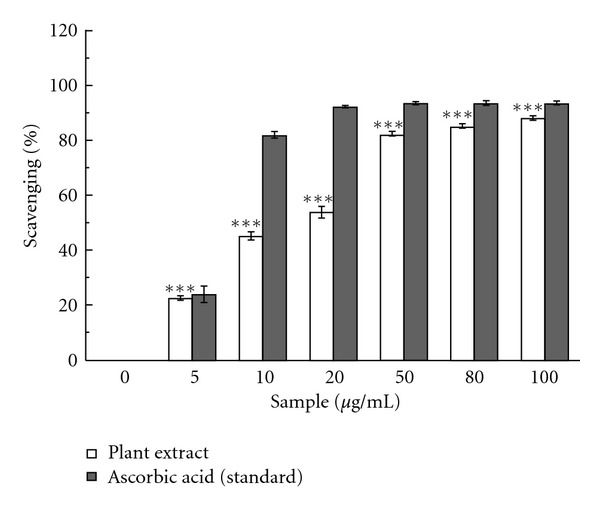
DPPH Radical Scavenging. DPPH radical scavenging activities of the CCME and the reference compound ascorbic acid. The data represent the percentage of scavenging of DPPH radical. The results are mean ± S.D. of six parallel measurements. ***P < 0.001 versus 0 *μ*g/ml.

The bleaching of DPPH indicates the free radical scavenging capacity of CCME.  Figure 1 demonstrated the DPPH radical scavenging activity of the extract in comparison to the standard. The IC_50_ values of the sample and standard were 14.2 ± 0.63 *μ*g/ml and 5.27 ± 0.27 *μ*g/ml, respectively. The results clarify the antioxidant activity of CCME as it is an effective DPPH free radical scavenger.

#### 3.1.2. DNA Damage

**Figure 2:**
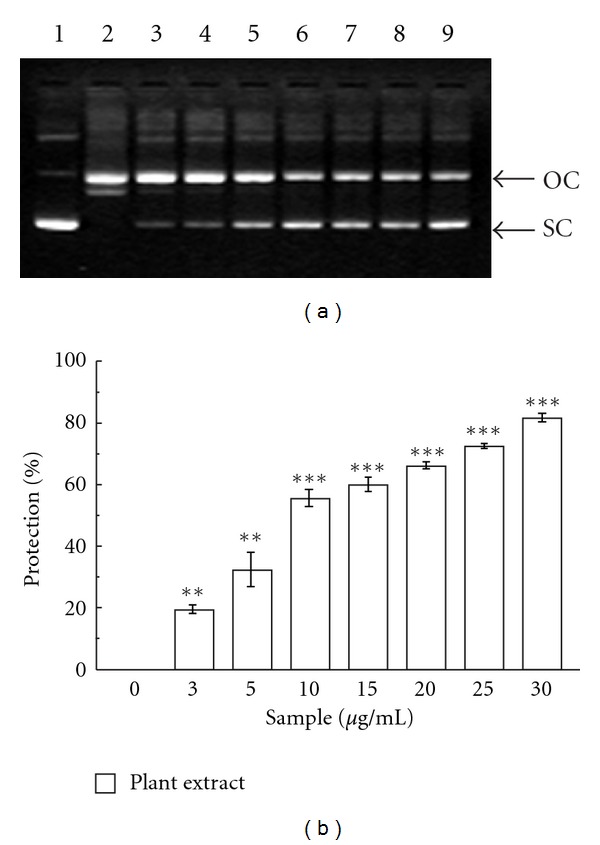
DNA protection. Protection against oxidative damage to pUC18 by CCME. Picture of agarose gel of pUC18 DNA showing bands of supercoiled (SC) and open circular (OC) forms. Lanes on the gel represent (lane 1) control DNA (no H_2_O_2_ or Fe^2+^); (lane 2) reaction mixture without extract; (lane 3–9) reaction mixture with extract of increasing concentration (3–30 *μ*g/ml). **P < 0.01 and ***P < 0.001 versus 0 *μ*g/ml.

The protective effect of CCME against Fe^2+^-dependent oxidative DNA damage of pUC18 plasmid was demonstrated in Figure 2. The results showed the dose-dependent protection of extract with a [P]_50_ value of 9.44 ± 0.76 *μ*g/ml. The significant reduction in the formation of nicked DNA and increase in supercoiled DNA in the presence of the CCME reveals its excellent iron-chelating activity.

### 3.2. *In Vivo* Study

#### 3.2.1. Serum Markers

The serum enzymes are very important adjuncts to clinical diagnosis of diseases and tissue injury. Hepatic injury by iron results in the leakage of cellular enzymes into the bloodstream, resulting in augmented levels of serum ALAT, ASAT, ALP, and bilirubin [4]. Increase in the levels of serum enzymes, namely, ALAT (136.24%), ASAT (145.69%), ALP (149.21%), and billirubin (265.48%) as shown in Table 1 clearly signifies iron-induced liver damage. Oral administration of CCME markedly reduced the elevated levels of serum enzymes and billirubin of iron-overloaded mice to approach the normal control values.

#### 3.2.2. Antioxidant Enzymes

Cells are armed with a stock of endogenous antioxidant defence machinery to protect them. These include enzymes such as SOD, CAT, and GST or compounds such as GSH [49]. In excess, iron is a major cause of oxidative stress and lowers the levels of SOD (77.04%), CAT (52.25%), GST (70.02%), and GSH (32.36%), whereas treatment with CCME arrested the iron-induced depletion of these enzymes dose-dependently (Table 2). Thus, CCME significantly mended the levels of antioxidant enzymes and helping revival from hepatic damage.

#### 3.2.3. Biochemical Parameters

**Figure 3:**
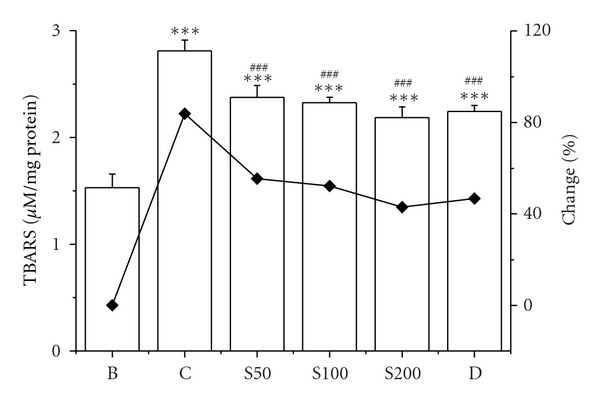
Lipid peroxidation. Hepatic lipid peroxidation levels in different treated mouse liver. Mice were randomly divided into six groups (blank, B; control, C; 50 mg/kg b.w. CCME, S50; 100 mg/kg b.w. CCME, S100; 200 mg/kg b.w. CCME, S200; desirox group, D) and treated as described in Section 2.6.1. Values are expressed as mean ± SD of six mice. ***P < 0.001 compared with blank and ^###^ *P* < 0.001 compared with control.

**Figure 4:**
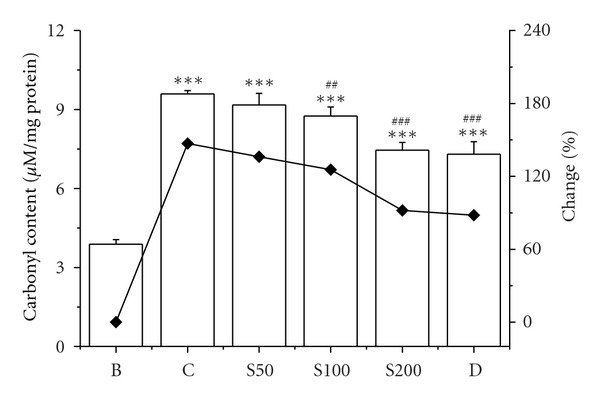
Protein oxidation. Inhibitory effect of CCME on protein oxidation levels in iron-overloaded mice. Mice were randomly divided into six groups (blank, B; control, C; 50 mg/kg b.w. CCME, S50; 100 mg/kg b.w. CCME, S100; 200 mg/kg b.w. CCME, S200; desirox group, D) and treated as described in Section 2.6.1. Protein carbonyl content was assayed to measure the extent of protein oxidation. Values are expressed as mean ± SD (*n* = 6). ***P < 0.001 compared with blank and ^##^ *P* < 0.01, ^###^ *P* < 0.001 compared with control.

**Figure 5:**
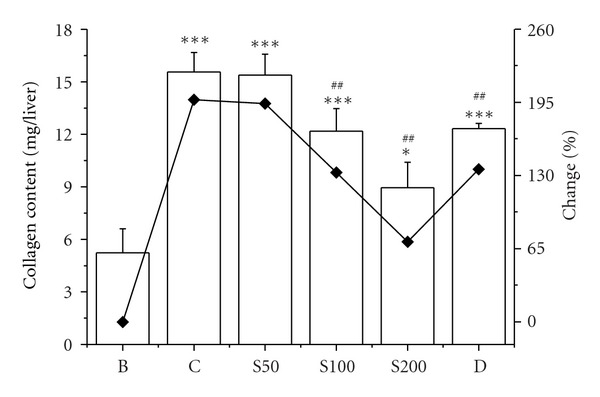
Collagen content. Collagen contents in different treated mouse liver. Mice were randomly divided into six groups (blank, B; control, C; 50 mg/kg b.w. CCME, S50; 100 mg/kg b.w. CCME, S100; 200 mg/kg b.w. CCME, S200; desirox group, D) and treated as described in Section 2.6.1. Hydroxyproline content was assayed to determine the liver fibrosis. Values are expressed as mean ± SD (*n* = 6). *P < 0.05, ***P < 0.001 compared with blank and ^##^ *P* < 0.01 compared with control.

The enhanced lipid peroxidation has been proposed as an initial step by which iron causes structural and functional alterations in cell integrity [50]. The present result showed that 84% increment of lipid peroxidation in liver homogenates of iron-injected mice than blank was significantly reduced by 29%, 32%, and 41% in mice fed with S50, S100, and S200, respectively, (Figure 3). Protein oxidation is another outcome in iron-overload-induced hepatic damage. The iron-mediated oxidative modification of protein leads to their degradation and carbonyl formation [51], which was confirmed by elevated level of protein carbonyl (147%) from liver samples in iron-overloaded mice compared to normal mice. The results of current study clearly establish that CCME efficiently reduced the carbonyl content 11%, 20%, and 55% with gradual increase of concentration (S50, S100, and S200) (Figure 4). The increase in collagen content (197%) of iron-intoxicated mouse was a significant indicator of liver fibrosis. The excessive iron deposition in liver resulted in iron-catalysed oxidative stress contributing to the pathogenesis and progression of liver fibrosis [52]. The collagen content in iron-overloaded mice was found 15.56 ± 1.09 mg/liver compared to 5.23 ± 1.37 mg/liver of normal mice. The upsurge of collagen content was gradually reduced to 15.38 ± 1.2 mg/liver, 12.17 ± 1.29 mg/liver, and 8.95 ± 1.45 mg/liver in CCME treated mice (S50, S100, and S200, resp.), signifying the hepatic fibrosis inhibitory potency of the plant extract (Figure 5).

#### 3.2.4. Histopathological Study

**Figure 6:**
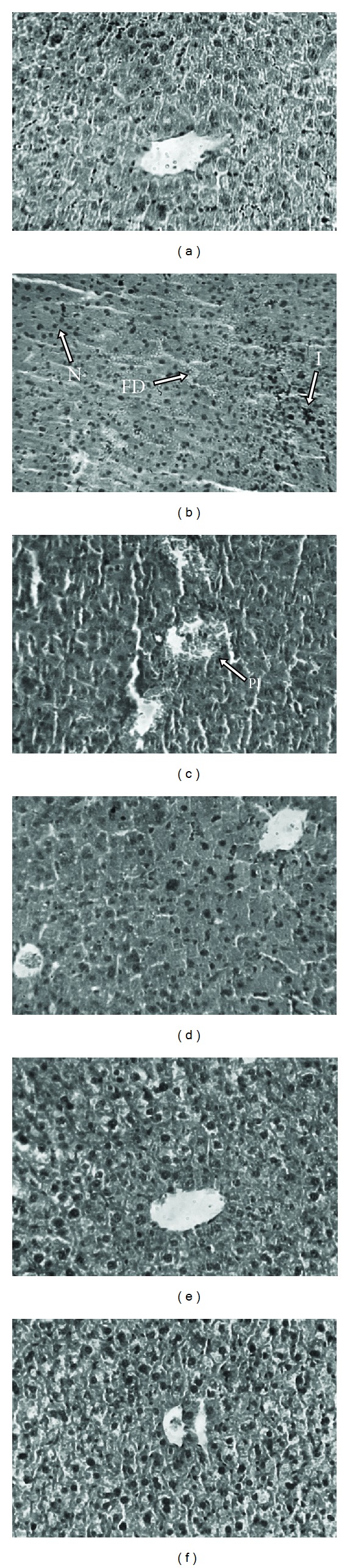
Histopathology. Photomicrograph of mice liver sections (staining with haematoxylin and eosin) ×40; (a) normal mice liver. (b) Iron-intoxicated (iron dextran, 100 mg/kg b.w.) liver section showing necrosis (N), fatty ballooning degeneration (FD), inflammation (I), and loss of cellular boundaries. (c) Liver section treated with iron dextran +50 mg/kg b.w. CCME showing improved histology with portal inflammation (PI). (d) Liver section treated with iron dextran +100 mg/kg b.w. CCME. (e) Liver section treated with iron dextran +200 mg/kg b.w. CCME. The two groups (d) and (e) show reduced hepatocellular necrosis, ballooning degeneration, and inflammation. (f) Liver section treated with iron dextran +20 mg/kg b.w. desirox also shows reduced necrotic area and increased number of hepatocytes.

Histological observations are performed along with the level of various biochemical parameters in circulation to mark the extent of hepatic damage. The liver sections of normal mice showed normal cell morphology with well-preserved cytoplasm, prominent nucleus, and well-brought-out central vein (Figure 6(a)). Iron dextran control mice showed various degrees of pathological changes including hepatocellular necrosis, ballooning degeneration, and loss of cellular boundaries (Figure 6(b)). In contrast, the liver sections taken from CCME-treated mice showed lessening of the pathogenesis and revealed marked reduction in hepatic injuries (Figures 6(c), 6(d), and 6(e)).  Figure 6(f) exhibited the improved histology of liver sections taken from desirox-treated group. However, these observations indicate the *in situ* hepatoprotective evidence of the extract.

#### 3.2.5. Liver Iron and Serum Ferritin

**Figure 7:**
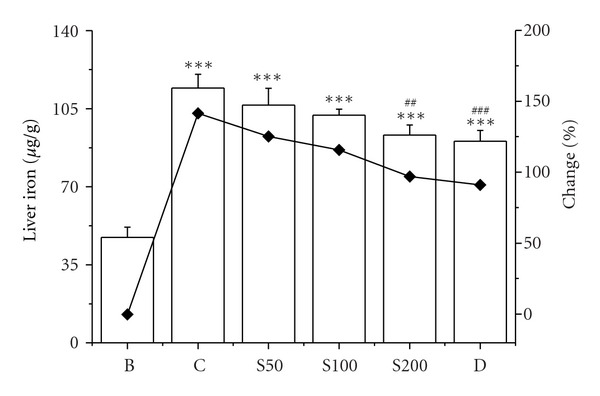
Liver iron. Effect of CCME on hepatic iron content in different treated mouse liver. Mice were randomly divided into six groups (blank, B; control, C; 50 mg/kg b.w. CCME, S50; 100 mg/kg b.w. CCME, S100; 200 mg/kg b.w. CCME, S200; desirox group, D) and treated as described in Section 2.6.1. Values are expressed as mean ± SD of six mice. ***P < 0.001 compared with blank and ^##^ *P* < 0.01, ^###^ *P* < 0.001 compared with control.

**Figure 8:**
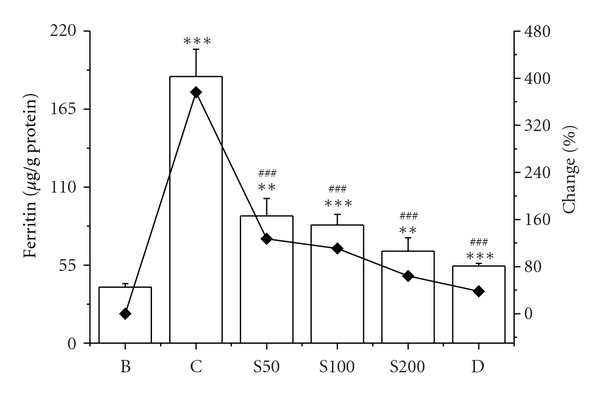
Serum ferritin. Serum ferritin levels in different treated mouse. Mice were randomly divided into six groups (blank, B; control, C; 50 mg/kg b.w. CCME, S50; 100 mg/kg b.w. CCME, S100; 200 mg/kg b.w. CCME, S200; desirox group, D) and treated as described in Section 2.6.1. Serum ferritin levels were assayed to demonstrate the degrees of iron overload. Values are expressed as mean ± SD of six mice. **P < 0.01, ***P < 0.001 compared with blank and ^###^ *P* < 0.001 compared with control.

The elevated (142%) liver iron content was found in iron-overloaded mouse compared to normal mouse. Administration of CCME reduced the iron level 17%, 26%, and 45% with the effect of increased dose (S50, S100, and S200, resp.) (Figure 7). The decrease in liver iron deposition induced by CCME treatment supports its iron-chelating potency which was established previously [35]. Body's iron level is positively correlated with ferritin, a ubiquitous intracellular protein that stores iron in a nontoxic form and also helps prevent iron from mediating oxidative damage to cell constituents [53]. The increased level of ferritin is generally noticed in iron-overload-induced liver toxicity which substantially reduced as treated with CCME dose-dependently (Figure 8).

#### 3.2.6. Reductive Release of Ferritin Iron and Its Correlation with Reducing Power

**Figure 9:**
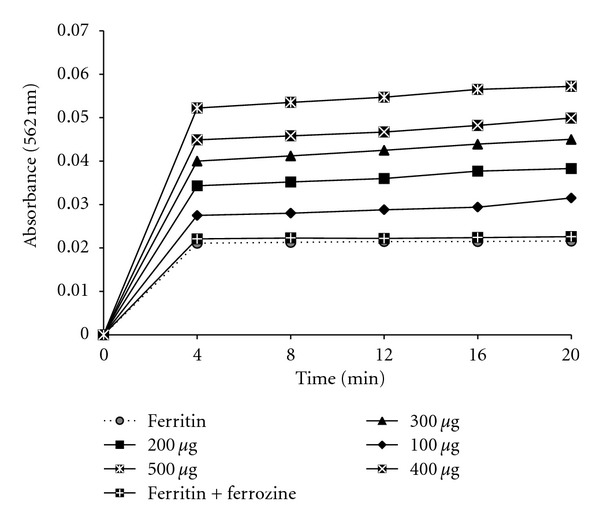
Dose-dependent formation of the [Fe(ferrozine)_3_]^2+^ complex following release of Fe^2+^ from ferritin by CCME with time. The reductive release of ferritin iron was quantified by measuring the formation of the ferrous complex of ferrozine, [Fe(ferrozine)_3_]^2+^ at 562 nm using a Shimadzu UV-VIS spectrophotometer.

**Figure 10:**
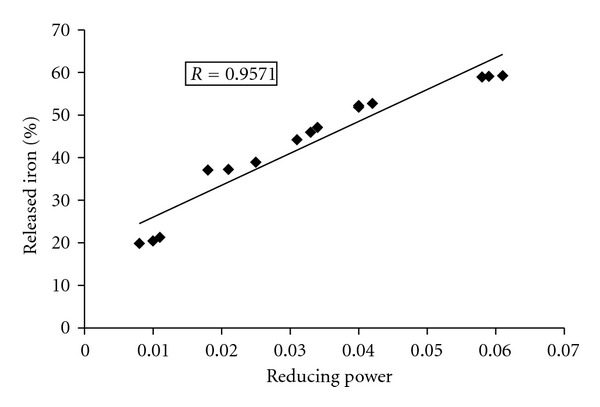
Correlation of reducing power versus release of ferritin iron (%) by CCME.

Within cell, ferritin served as the storage protein for excess iron in ferric state. In case of iron overload, various iron chelators are administered to attenuate the situation, but, most of these chelators have limited binding activity for ferric iron as well, as iron in ferritin is not properly accessed to them. So, iron chelation therapy is dependent on the reductive release of ferritin iron, which is achieved by supplemented addition of a reducing agent such as ascorbate to increase the availability of storage iron to chelators [54].  Figure 9 showed the reductive release of ferritin iron by CCME, that was measured with a ferrous complex of ferrozine [Fe(ferrozine)_3_]^2+^. Control experiments without CCME produced negligible amounts of [Fe(ferrozine)_3_]^2+^, whereas, after dose dependant addition of CCME the [Fe(ferrozine)_3_]^2+^ complex formation was increased significantly with time. However, reducing property of an iron chelator should definitely increase the efficiency of iron chelation therapy to treat iron overload. Previous results had shown the reductive ability of CCME [35] as well as in the present study, a significant (*P* < 0.001) positive correlation (*R* = 0.9571) between reducing power and (%) iron released from ferritin has been well established (Figure 10).

## 4. Conclusions

From the present study, it might be concluded that CCME has protective effect against iron-overload-induced liver toxicity as evidenced by biochemical and histopathological studies. CCME may exert its hepatoprotective activity by upregulating antioxidant enzymes and chelating iron to excrete form the body. The findings suggest its benefit in pathological sequence of iron-overload-linked liver disease and can be used as promising hepatoprotective agent.

## Figures and Tables

**Table 1:** The effect of CCME on serum marker enzymes (ALAT, ASAT, and ALP) and billirubin in iron overloaded mice.

Treatment	ALAT		ASAT		ALP		Billirubin	
	Unit/L	% change	Unit/L	% change	Unit/L	% change	Unit/L	% change
B	19.32 ± 1.85	—	28.18 ± 1.48	—	143.49 ± 5.76	—	1.16 ± 0.05	—
C	45.64 ± 4.26^X2^	136.24	69.25 ± 7.61^X2^	145.69	357.59 ± 12.45^X3^	149.21	4.25 ± 0.58^X2^	265.48
S50	38.35 ± 3.37^X2Y2^	98.5	56.74 ± 6.18^X2Y1^	101.3	353.11 ± 20.01^X3^	146.08	4.22 ± 0.4^X2^	262.53
S100	36.08 ± 1.17^X2Y1^	86.74	53.95 ± 4.84^X1Y3^	91.39	338.43 ± 10.85^X2^	135.84	3.98 ± 0.24^X2^	241.88
S200	34.86 ± 1.49^X3Y1^	80.42	51.95 ± 1.38^X3Y1^	84.31	271.26 ± 24.43^X2Y1^	89.04	3.27 ± 0.26^X2Y1^	180.82
D	23.55 ± 1.39^X2Y2^	21.88	44.68 ± 5.52^X1Y2^	58.49	151.58 ± 9.92^Y3^	5.63	1.60 ± 0.12^X2Y1^	37.16

B: Normal mice; C: iron overload control; S50: iron overload + CCME (50 mg/kg b.w.); S100: iron overload + CCME (100 mg/kg b.w.); S200: iron overload + CCME (200 mg/kg b.w.); D: iron overload + desirox (20 mg/kg b.w.).

Values are mean ± SD of six observations.

X: significant difference from normal mice (B) group (X1: *P* ≤ 0.05; X2: *P* ≤ 0.01 and X3: *P* ≤ 0.001).

Y: significant difference from iron overloaded (C) group (Y1: *P* ≤ 0.05; Y2: *P* ≤ 0.01; Y3: *P* ≤ 0.001).

**Table 2:** The effect of CCME on antioxidant enzymes (SOD, CAT, and GST) and nonenzymatic antioxidant, GSH in iron overloaded mice.

Treatment	SOD		CAT		GST		GSH	
	Unit/mg protein	% change	Unit/mg protein	% change	Unit/mg protein	% change	*μ*g/mg protein	% change
B	0.62 ± 0.05	—	21.55 ± 2.24	—	7.19 ± 0.71	—	0.48 ± 0.01	—
C	0.14 ± 0.07^X3^	77.04	10.29 ± 0.73^X3^	52.25	2.16 ± 0.51^X3^	70.02	0.32 ± 0.01^X3^	32.36
S50	0.26 ± 0.04^X3Y1^	58.58	10.59 ± 0.34^X3^	50.83	3.49 ± 0.67^X3Y2^	51.45	0.42 ± 0.02^X3Y3^	10.85
S100	0.31 ± 0.07^X3Y2^	50.72	11.81 ± 0.71^X3Y1^	45.21	4.87 ± 0.57^X3Y3^	32.21	0.45 ± 0.02^Y3^	5.63
S200	0.32 ± 0.05^X3Y1^	48.95	15.14 ± 0.77^X2Y3^	29.7	4.98 ± 0.68^X1Y2^	30.77	0.46 ± 0.01^X1Y3^	3.55
D	0.39 ± 0.06^X3Y2^	36.59	18.97 ± 0.81^X1Y3^	11.97	3.13 ± 0.53^X3Y1^	56.47	0.39 ± 0.05^X3Y3^	18.37

B: Normal mice; C: iron overload control; S50: iron overload + CCME (50 mg/kg b.w.); S100: iron overload + CCME (100 mg/kg b.w.); S200: iron overload + CCME (200 mg/kg b.w.); D: iron overload + desirox (20 mg/kg b.w.).

Values are mean ± SD of six observations.

X: significant difference from normal mice (B) group (X1: *P* ≤ 0.05; X2: *P* ≤ 0.01 and X3: *P* ≤ 0.001).

Y: significant difference from iron overloaded (C) group (Y1: *P* ≤ 0.05; Y2: *P* ≤ 0.01; Y3: *P* ≤ 0.001).
